# Morphological Characteristics of Maize Canopy Development as Affected by Increased Plant Density

**DOI:** 10.1371/journal.pone.0154084

**Published:** 2016-04-29

**Authors:** Youhong Song, Yukui Rui, Guta Bedane, Jincai Li

**Affiliations:** 1 Anhui Agricultural University, School of Agronomy, Hefei, Anhui Province, 230036, China; 2 China Agricultural University, College of Resource and Environmental Sciences, Beijing, 100094, China; 3 The University of Queensland, School of Agriculture and Food Sciences, Gatton, Qld, 4343, Australia; 4 Jiangsu Collaborative Innovation Center for Modern Crop Production, Nanjing, 210095, China; University of Delhi, INDIA

## Abstract

Improving crop productivity through higher plant density requires a detailed understanding of organ development in response to increased interplant competition. The objective of this paper is thus to investigate the characteristics of organ development under increased interplant competition. A field experiment was conducted to investigate organ development across 4 maize plant densities i.e. 2, 6, 12 and 20 plants m^–2^ (referred to PD2, PD6, PD12 and PD20 respectively). In response to increased interplant competition, lengths of both laminae and sheaths increased in lower phytomers, but decreased in upper phytomers. Sheath extension appeared to be less sensitive to increased interplant competition than lamina extension. Extension of laminae and internodes responded to increased plant density as soon as onset of mild interplant competition, but did not respond any further to severe competition. Both lamina width and internode diameter were reduced due to a smaller growth rate in response to increased plant density. Overall, this study identified that organ expansion rate can be taken as the key morphological factor to determine the degree of interplant competition.

## Introduction

Optimising high plant density may increase the potential for achieving greater crop yield since there are more plants available per single harvesting unit (e.g. per hectare). Nevertheless, harvest of crop biomass and grain yield can decline if plant density exceeds certain thresholds [1,2,3] though modern hybrids have an improved capacity to withstand high plant density [4]. Accordingly, the optimisation of plant density is of great interest in the maximisation of maize productivity for different hybrids that continue to be developed in agricultural practice [2, 5, 6, 7]. Crop growth and yield in maize depends on incident radiation intercepted by the canopy [8,9] and light composition of intercepted radiation such as Red (R): Far Red (FR) [10,11]. Both intercepted light quantity and its quality in the canopy are largely determined by canopy structure, which is influenced by plant population density [12, 13, 14]. Consequently, much attention has been directed to how the maize canopy responds to increased plant density [12, 14, 15].

The effects of increased plant density on maize morphological development have been examined extensively at the canopy level including plant height, leaf area per plant and leaf area index [7, 12, 15, 16, 17, 18]. It should be noted that the whole plant level effects are realised through organ responses that may vary with positions in different types of organs [19, 14, 20]. For example, the effect of plant density on leaf size differs in leaf width and length, the former being consistently reduced in both lower and upper phytomers [21] whereas the latter being increased in lower phytomers and reduced in upper phytomers [22]. Hence, it is necessary to focus on organ morphological response to increased plant density for better understandings of the crop genetic basis that may be neglected at the whole canopy level with respect to intraspecific competition.

Therefore the objective of this paper is to investigate canopy morphological characteristics in response to increased interplant competition by examining the effects of increased plant density on development of individual laminae, sheaths and internodes.

## Materials and Methods

### Cultural details

The field experiment, in combination with another field experiment with different water regimes, was described briefly in deriving relationships between organ morphology and biomass [23]. Here we would describe the experimental details associated with the effects of increased interplant competition on organ development for this paper. The field experiment of maize grown under four plant densities i.e. 2, 6, 12 and 20 plants m^–2^ (referred to as PD2, PD6, PD12 and PD20 respectively) was undertaken at Shangzhuang experimental station of China Agricultural University, Beijing, China (Latitude 40°02’N, Longitude 116°20’E). The maize hybrid investigated was ‘NongDa 108’ (referred to as ND108). The soil type in the field site was a light loam. Prior to sowing, an initial irrigation was applied at 900 cm^3^ ha^–1^ and base nutrients were used at 60 kg ha^–1^ for N, at 135 kg ha^–1^ for P_2_O_5_, at 120 kg ha^–1^ for K_2_O, and at 15 kg ha^–1^ for ZnSO_4_.7H_2_O. Seeds were evenly sown manually in each row on July 7^th^, 2009. The seedlings were then thinned to the target plant densities (see the experimental design below) during the third leaf stage in each plot. Subsequent irrigation, depending on rainfall, was applied to prevent canopy development from water stress. The weeds in the field were removed manually.

### Experimental design

A randomized complete block design of four plant densities was used, each plant density having three replicates. The lowest density of PD2 represented no or mild interplant competition, used as the reference; the treatment of PD6, closing to local farming practice, represented the moderate interplant competition; the treatment of PD12 represented severe interplant competition; and the treatment of P20 represented extremely severe interplant competition. The four levels of plant population allowed us to generate a large variation of interplant competition in order to demonstrate the effects of increased plant density. The rows were 75 cm apart in the first three treatments and 50 cm apart in the PD20. Each plot was 7 m long by 6 m wide. Plots within a block were separated by a 1 m bare space and independent block were separated by a 2 m bare space. The 4 outside edges of the whole planting area were bordered by two rows of extra guarding plants.

### Data collection

Ten reference plants in each plot were chosen to guide destructive samplings of canopy development when plants had 5 visible leaves (3 fully expanded). During the selection, thirty plants were chosen randomly in the plot (excluding the edge row), the visible lengths of leaves 3 and 4 of those plants were then measured and averaged. Ten continuous plants in a row with lengths of leaves 3 and 4 closest to average values of these leaves on the thirty plants were marked as reference plants for each plot. Leaf 5, 10, and 15 in 10 reference plants were respectively labeled when each of them was fully expanded, in order to assist to judge the leaf number for samples. Destructive sampling commenced from the 4^th^ fully expanded leaf to canopy expansion completion at 1–2 day intervals. Prior to each destructive sampling, plant height, number and lengths of expanding leaves on reference plants were measured to provide a quantitative basis on which to choose single plants in each plot. The plants once destructively sampled were immediately brought to the laboratory and dissected as individual organs for measurement. The rank of each phytomer in the plant was counted acropetally. Leaves in the lower positions may have dropped off due to leaf senescence, so the existing leaf number in each sample is determined by retrieving closest leaf length and width with that of the specific leaf in reference plants. Data on total and fully expanded (indicated by presence of ligule) leaf number, lamina length, lamina maximum width (referred to as lamina width), sheath length and internode length and diameter (once stem elongation started) for each phytomer were measured during each destructive sampling. Data on daily minimum and maximum temperatures were collected from a weather station near the field site.

### Data analyses

Modern maize hybrids barely produce tillers even at low plant density [21], which were rarely observed in any plant density of this study. Thus the analyses of plant data were based on the sampled plants without any tillers. Organ development was related to the accumulated thermal time after emergence (°Cd AE) using 8°C as base temperature [24]. Data on organ lengths used to derive time course of organ extension were the average values of organ length in corresponding phytomer position from three replicates in each treatment. Final size of individual organs was calculated by averaging the data at the corresponding position collected after the organ was fully expanded, and standard errors were also determined. The difference (%) was then calculated as (PD20-PD2)/PD2*100% if the difference between treatments was significant. To visually illustrate the effect of interplant competition on organ development more clearly, only two treatments i.e. PD2 and PD20 with largest difference were chosen to represent. The rate of extension of organs was estimated as final length divided by the duration of extension and the standard deviation was at 95% confidence interval. Lengths of first 6 internodes were very short and not presented here. The internodes from phytomer 15 onwards were not accounted since there are insufficient repetitive sampling data for calculation of internode length after internodes are fully expanded. Statistical analyses on whether final size of individual leaves, sheaths and internodes (repeats for mean values equal or over 3 samples according to available data) were affected by increased plant density were carried out using paired two-sample t-test provided by Microsoft Excel 2007 (Microsoft Inc., Seattle, WA, USA) at significant level of 0.05.

## Results

### Organ final size to increased plant density

#### Lamina final length

**Fig 1:**
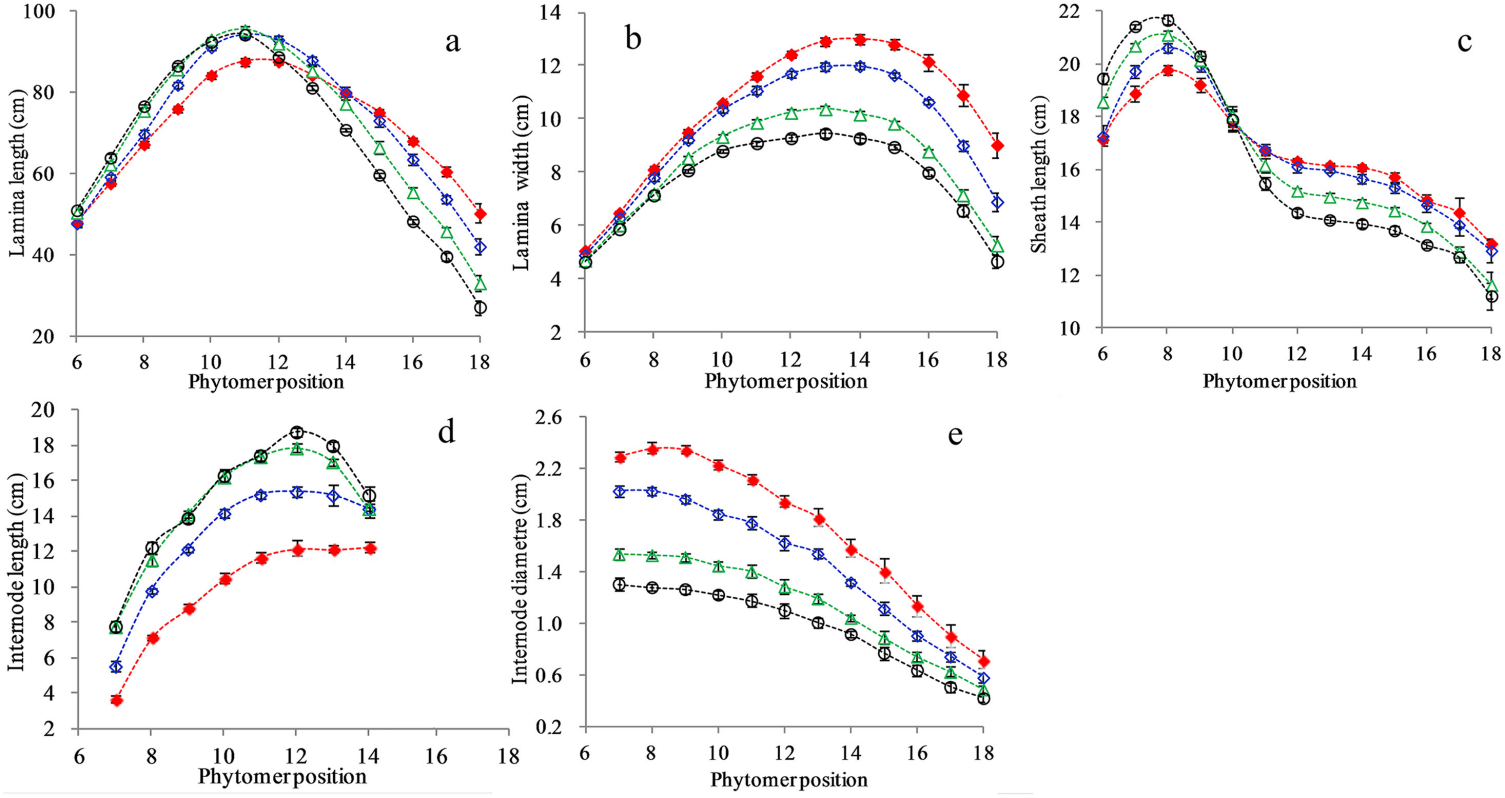
Lamina length (a), lamina width (b), sheath length (c), internode length (d) and internode diameter (e) in PD2 (red filled diamond), PD6 (blue open diamond), PD12 (green open triangle) and PD20 (black open circle) as a function of phytomer position [x axis starts from phytomer 6 to 18; y axis starts from 20 cm for (a), from 2 cm for (b), from 10 cm for (c), from 2 cm for (d) and from 0.2 cm for (e); and vertical bars indicate standard errors].

Lamina length was significantly increased by 7.7, 8.2, 7.6, 6.1 and 4.4% for phytomers 9–13, respectively when plant density increased from PD2 (red dash line) to PD6 (blue dash line) (Fig 1a), and by 8.1 and 4.6% for phytomers 8 and 9, respectively when plant density continued to increase from PD6 (blue dash line) to PD12 (green dash line) (Fig 1a). However there were no phytomers responded when plant density further increased from PD12 (green dash line) to PD20 (black dash line) (Fig 1a). The results indicated that lamina extension in lower and middle phytomers quickly responded to mild and moderate interplant competition, but arrested to respond further to more severe competition.

Lamina length was significantly reduced by 6.6, 11.1 and 16.2% for phytomers 16–18 respectively when plant density increased from PD2 (red dash line) to PD6 (blue dash line) (Fig 1a); by 9.1, 12.7, 14.8 and 21.5% for phytomers 15–18 respectively when plant density continued to increase from PD6 (blue dash line) to PD12 (green dash line) (Fig 1a); and by 3.5, 4.8, 8.2, 10.1, 12.8, 13.4 and 17.6% for phytomers 12–18 respectively when plant density further increased from PD12 (green dash line) to PD20 (black dash line) (Fig 1a). The results indicated that the effect of increased plant density on upper laminae continued as interplant competition elevated from mild to severe interplant competition.

As a consequence, there were two types of effects on lamina length induced by interplant competition due to increased plant density: i) increase in lower and middle phytomers and ii) reduction in upper phytomers.

#### Lamina final width

Lamina width was significantly reduced by 5, 6, 7, 8, 9, 12, 17 and 24% for phytomers 11–18 respectively when plant density increased from PD2 (red dash line) to PD6 (blue dash line) (Fig 1b); by 8, 7, 10, 11, 13, 13, 15, 16, 17, 21 and 24% for phytomers 8–18 respectively when plant density continued to increase from PD6 (blue dash line) to PD12 (green dash line) (Fig 1b); and by 5, 6, 8, 9, 9, 9, 9, 9, 8 and 11% for phytomers 9–18 respectively when plant density further increased from PD12 (green dash line) to PD20 (black dash line) (Fig 1b). Consequently, leaf width tended to become narrower and narrower as interplant competition elevated from mild to severe interplant competition.

#### Sheath final length

Sheath length was increased by 3.0, 4.2 and 3.8% for phytomers 7–9 respectively when plant density increased from PD2 (red dash line) to PD6 (blue dash line) (Fig 1c); by 7.5 and 4.8% for phytomers 6–7 respectively when plant density continued to increase from PD6 (blue dash line) to PD12 (green dash line) (Fig 1c); and by 4.8% only for phytomer 6 when plant density further increased from PD12 (green dash line) to PD20 (black dash line) (Fig 1c). The result showed that only a few sheaths in lower phytomers have responded to increased plant density and there were fewer phytomers responded as interplant competition continued to increase.

Extension in upper sheaths was not affected from PD2 (red dash line) to PD6 (blue dash line) (Fig 1c). However sheath length was reduced by 5.7, 6.2, 5.7, 5.7 and 5.5% for phytomers 12–16 respectively when plant density continued to increase from PD6 (blue dash line) to PD12 (green dash line) (Fig 1c); and reduced by 4.3, 5.4, 5.8, 5.5, 5.2 and 5.1% for phytomers 11–16 respectively when plant density further increased from PD12 (green dash line) to PD20 (black dash line) (Fig 1c). Overall, sheath extension was not responsive to interplant competition until PD6 was surpassed; the reduction rate for those phytomers affected by increased interplant competition was relatively low; and the effect continued as interplant competition further increased.

#### Internode final length

Internode length was significantly increased by 49.0, 36.1, 37.5, 34.8, 29.7, 26.2, 24.9 and 17.4% for phytomers 7–14 respectively when plant density increased from PD2 (red dash line) to PD6 (blue dash line) (Fig 1d), the response extent declining as phytomer increased; by 40.6, 18.2, 16.3, 14.5, 14.2, 16.2 and 12.5% for phytomers 7–13 respectively when plant density increased from PD6 (blue dash line) to PD12 (green dash line) (Fig 1d), the response extent decreasing as phytomer increased; but it was not increased any further from PD12 (green dash line) to PD20 (black dash line) (Fig 1d). Overall, internodes quickly responded to mild and moderate interplant competition with a great extent, but the effect completely arrested beyond severe interplant competition. The response extent of internode elongation to increased plant density decreased as the phytomer increased, suggesting that lower internodes were more sensitive to increased interplant competition than upper internodes.

#### Internode final diameter

Internode final diameter was significantly reduced by 11.7, 14.3, 16.4, 17.2, 16.1, 16.5, 15.5, 16.8, 20.9, 20.6, 18.3 and 19.5% for phytomers 7–18 respectively from PD2 (red dash line) to PD6 (blue dash line) (Fig 1e); by 24.0, 24.4, 22.8, 21.6, 20.9, 20.8, 22.4, 20.8, 20.1, 17.9 and 15.8% for phytomers 7–17 respectively from PD6 (blue dash line) to PD12 (green dash line) (Fig 1e); and by 15.1, 16.2, 16.3, 15.5, 16.2, 14.2, 15.3, 12.0, 13.1, 14.0 and 18.4% for phytomers 7–17 respectively from PD12 (green dash line) to PD20 (black dash line) (Fig 1e). Overall, internode diameter expansion quickly responded to onset of interplant competition and the response remained when interplant competition was beyond severe level.

### Organ extension in response to increased plant density

#### Lamina extension

**Fig 2:**
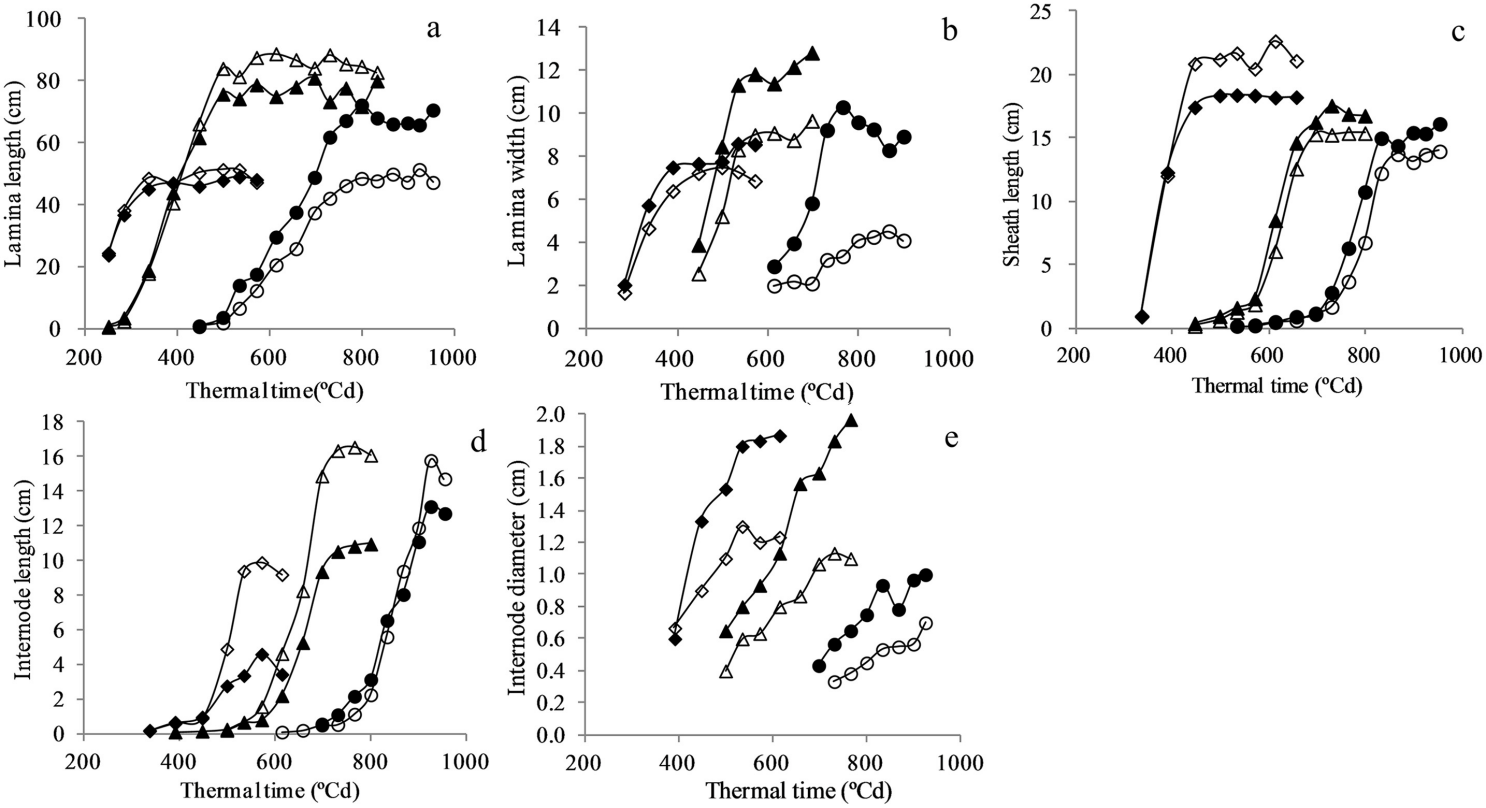
Lamina extension (a), lamina width expansion (b), sheath extension (c), internode extension (d) and internode diameter expansion (e) against thermal time after emergence in PD2 (filled symbol) and PD20 (open symbol) [exampled as phytomer 6 (diamond), 9 (triangle) and 16 (circle) for (a); phytomer 8 (diamond), 12 (triangle) and 18 (circle) for (b); phytomer 7 (diamond), 11 (triangle) and 15 (circle) for (c); phytomer 7 (diamond), 10 (triangle) and 14 (circle) for (d); phytomer 7 (diamond), 11(triangle) and 16 (circle) for (e); x axis starts from 200°Cd].

To investigate the reason for the effect of increased plant density on lamina final length, time course of lamina extension in three representative phytomers 6 (diamond), 9 (triangle) and 16 (circle) in PD2 (filled symbol) and PD20 (open symbol) is presented (Fig 2a). Lamina extension in lower phytomers was promoted by increased plant density while lamina extension in upper phytomers was reduced. The increased lamina length in lower phytomers was due to a greater rate of extension [the extension rate is 0.238±0.0119 cm°Cd^-1^ (phytomer 6), 0.302±0.0151 cm°Cd^-1^ (phytomer 9) in PD2 compared to 0.286±0.0143 cm°Cd^-1^ (phytomer 6), 0.337±0.0168 cm°Cd^-1^ (phytomer 9) in PD20, visually see Fig 2a] while the reduced lamina length in upper phytomers was due to a smaller rate of extension [0.203±0.0101 cm°Cd^-1^ in PD2 compared to 0.136±0.0068 cm°Cd^-1^ (phytomers 16), visually see Fig 2a].

#### Lamina width expansion

To investigate the reason for the effect of increased plant density on lamina final width, time course of lamina width growth in three representative phytomers 8 (diamond), 12 (triangle), 18 (circle) in PD2 (filled symbol) and PD20 (open symbol) is presented (Fig 2b). Lamina width was consistently reduced for phytomer 8 onwards when plant density increased from PD2 to PD20. The reduction of lamina width can be attributed to a smaller growth rate [the growth rate is 0.051±0.0026 cm°Cd^-1^ (phytomer 8), 0.085±0.0043 cm°Cd^-1^ (phytomer 12), 0.046±0.0023 cm°Cd^-1^ (phytomer 18) in PD2 compared to 0.034±0.0017 cm°Cd^-1^ (phytomer 8), 0.052±0.0026 cm°Cd^-1^ (phytomer 12), 0.025±0.0012 cm°Cd^-1^ (phytomer 18) in PD20] though a slight delay was also observed in phytomer 18 (visually see Fig 2b).

#### Sheath extension

To investigate the reason for the effect of increased plant density on sheath final length, time course of sheath extension in three representative phytomers 7 (diamond), 11 (triangle) and 15 (circle) in PD2 (filled symbol) and PD20 (open symbol) is presented (Fig 2c). Data on sheath extension were insufficient for phytomers 16–18 due to rapid growth over the interval of sampling, so only phytomers under 16 are presented. In response to increased plant density, a similar effect to lamina extension were observed in sheath extension i.e. increase for lower phytomers while reduction for upper phytomers. The increase in sheath length for lower phytomers was due to a greater rate of extension [0.150±0.0075 cm°Cd^-1^ (phytomer 7) in PD2 compared to 0.181±0.0091 cm°Cd^-1^ (phytomer7) in PD20, visually see Fig 2c], while the reduction in sheath length for upper phytomers was due to a smaller rate of extension [the extension rate is 0.063±0.0032 cm°Cd^-1^ (phytomers 11) and 0.056±0.0028 cm°Cd^-1^ (phytomer 15), compared to 0.060±0.0030 cm°Cd^-1^ (phytomer 11) and, 0.046±0.0023 cm°Cd^-1^ (phytomer15), visually see Fig 2c].

#### Internode elongation

To investigate the reason for the effect of increased plant density on internode final length, time course of internode elongation in three representative phytomers 7 (diamond), 10 (triangle) and 14 (circle) in PD2 (filled symbol) and PD20 (open symbol) is presented (Fig 2d). Internode elongation was entirely promoted for phytomers 7–14 as plant density increased from PD2 to PD20 and the increase was primarily attributed to a greater rate of extension [the extension rate is 0.016±0.0008 cm°Cd^-1^ (phytomer 7), 0.044±0.0022 cm°Cd^-1^ (phytomer 10) and 0.055±0.0028 cm°Cd^-1^ (phytomer 14) in PD2, compared to 0.047±0.0023 cm°Cd^-1^ (phytomer 7), 0.070±0.0035 cm°Cd^-1^ (phytomer 10) and 0.067±0.0034 cm°Cd^-1^ (phytomer 14) in PD20, visually see Fig 2d]. For last few phytomers (15–18), internode elongation was not affected by increased plant density (data not shown).

#### Internode diameter expansion

To investigate the reason for the effect of increased plant density on internode final diameter, time course of internode diameter dynamics in three representative phytomers 7 (diamond), 11 (triangle) and 16 (circle) in PD2 (filled symbol) and PD20 (open symbol) is presented (Fig 2e). Internode diameter was entirely reduced for three phytomers as plant density increased from PD2 to PD20. The reduction in internode diameter was primarily attributed to the smaller growth rate [the growth rate is 0.0029±0.0001 cm°Cd^-1^, 0.0051±0.0003 cm°Cd^-1^ and 0.0036±0.0002 cm°Cd^-1^ in PD2 compared to 0.0012±0.0001 cm°Cd^-1^, 0.0032±0.0002 cm°Cd^-1^ and 0.0019±0.0001 cm°Cd^-1^ in PD20, visually see Fig 2e].

## Discussion

### Organ morphological characteristics under increased interplant competition

This study investigated organ development as affected by increased plant density and identified organ morphological characteristics in response to increased interplant competition.

Lamina extension response to increased plant density showed two different patterns i.e. increase for lower phytomers and reduction for upper phytomers, which is consistent with reports in [22]. We have used a range of plant densities i.e. 2, 6, 12 and 20 plants m^−2^ while two plant densities i.e. 9.5 and 30.5 plants m^−2^ were used in [22], constituting an extensive range from low plant density to extremely high plant density, in demonstrating the effects of increased plant density. The findings in [22] were based on two relatively high plant densities, so the intermediate response to sequential increase of plant density remains to be determined. Our data provided four levels of plant densities ranging from low to high interplant competition, identifying fine response characteristics to increased interplant competition. The increase of lamina length for those affected phytomers was attributed to a greater rate of extension while the reduction for those affected phytomers was attributed to a smaller rate of extension. A similar effect of increased plant density on leaf laminae was also observed in leaf sheaths, though there are differences between them. For lower phytomers, lamina and sheath extension response to increased plant density started as soon as PD2 density was surpassed, but lamina extension arrested when PD12 was surpassed (beyond severe competition) while sheath extension effects continued. For upper phytomers, lamina extension response to increased plant density started as soon as PD2 was surpassed while sheath extension started from PD6 on. The reduction for both laminae and sheaths in upper positions continued even when PD12 was surpassed (beyond severe competition). In addition, there were fewer sheaths affected and a smaller rate of either increase or decrease in sheath extension in response to increased plant density. This indicated that sheath extension was less sensitive to increased interplant competition than lamina extension.

In response to increased interplant competition, internode elongation was promoted due to a greater elongation rate. Promotion of both internode and lamina extension in response to increased interplant competition started as soon as PD2 density was surpassed, but arrested after PD12 was surpassed (beyond severe competition). It is noted that the effect on internode elongation were more significant compared to lamina in lower phytomers in response to increased plant density (Fig 1). Both lamina width and internode diameter were reduced in both lower and upper phytomers by interplant competition, and the effect took place as soon as PD2 was surpassed and even remained after PD12 (beyond severe competition). It is also noted that the effect on internode diameter expansion was far more evident compared to lamina width expansion in response to increased plant density (Fig 1).

It would be interesting to compare the different morphological responses of a collection of maize cultivars that have genetic variation for tolerance to high plant density. The morphological characteristics in response to increased plant density may be amenable to molecular design of plant ideotype for withstanding higher plant density.

### Physiological basis of organ response to increased interplant competition

Increased plant density causes changes of maize canopy development under elevated interplant competition. This is related to changes in light quantity [12, 14] and light quality (R: FR) [25] within the canopy. Increased internode elongation and leaf extension in response to interplant competition is linked to the detection of lower R: FR [10]. Greater internode length and leaf length lift up leaves in the lower canopy to receive more light. Leaf shape change (narrow leaves) allows more incident light to penetrate deeper into the lower canopy for photosynthesis. The decrease of lamina and sheath length in upper phytomers may be dominated by low carbohydrate availability. In response to increased interplant competition, leaf and internode promoted extension in sacrifice of width expansion. This suggests that leaf and stem adjusted cellular development in response to increased competition, which needs to be further explored by microscopic studies. The extent of internode elongation promotion in response to increased plant density decreased as the number of phytomers increased. The difference may be due to bottom internodes having received less radiation as canopy depth increased.

It should be noted that slimmer stems due to increased plant density often cause negative effects e.g. more susceptible to stalk lodging (observed in the experiment). As such, improving crop yield via higher plant density will be related to improvement of stalk lodging resistance. To such end, fundamental studies on the biological basis that underlies anatomical, physiological and biochemical mechanisms to increased interplant competition are worthy of more attention. The various responses of organ morphology to increased interplant competition provide a base to further investigate lodging resistance mechanisms under high plant density cropping.

## Conclusions

This study compared organ development across a range of plant densities and revealed maize canopy morphological characteristics at organ level in response to increased interplant competition. In adaption to increased plant density, the extension of both laminae and sheaths was promoted in lower phytomers due to a greater rate of extension and was reduced in upper phytomers due to a smaller rate of extension. However, the number of affected phytomers and extent of phytomers affected differed between laminae and sheaths. Extension of laminae and internodes responded to interplant competition as soon as mild competition was detected (e.g. PD2 was surpassed), nevertheless, the effect did not continue any further beyond severe competition (e.g. when PD12 was surpassed). Both lamina width and internode diameter were reduced due to a smaller expansion rate in response to interplant competition. As a consequence, the response of individual organs to increased plant density largely varied with organ types, positions and intensities of interplant competition, and the responses of various organs at different positions used extension/expansion rates to modify their morphological characteristics in adaption to increased interplant competition. Our findings revealed the necessity of further investigations on the physiological and biological basis of organ development in relation to high plant density.

## Supporting Information

S1 Dataset This excel file includes leaf lamina expansion time course in PD2, PD6, PD12 and PD20. The effects of increased plant density on lamina final size and expansion are based on this dataset.
(XLSX)

S2 Dataset This excel file includes leaf sheath time course in PD2, PD6, PD12 and PD20. The effects of increased plant density on sheath final size and extension are based on this dataset.
(XLSX)

S3 Dataset This excel file includes internode expansion time course in PD2, PD6, PD12 and PD20. The effects of increased plant density on internode final length and diameter and expansion are based on this dataset.
(XLSX)
